# Surgery assistance system for continuous resection of brain tumors-proposal of continuous tumor resection forceps, tumor cell separation, dehydration, and isolation mechanism

**DOI:** 10.1007/s11548-023-02845-x

**Published:** 2023-02-21

**Authors:** Taro Koguchi, Funika Shimizu, Tomohiro Nagame, Yuka Goto, Hikaru Iwasaki, Akihiko Hanafusa, Motoki Takagi, Shahrol Mohamaddan, Kenichi Nomura, Yoshihiro Muragaki, Hiroshi Iseki, Ken Masamune, Toyohisa Akitaya

**Affiliations:** 1 Department of Bio-Science and Engineering, Shibaura Institute of Technology, 307 Fukasaku, Minuma-ku, Saitama, 337-8570 Japan; 2 Cooperative Major in Advanced Biomedical Sciences, Joint Graduate School of Tokyo Women’s University and Waseda University, Tokyo, Japan; 3 Institute of Advanced BioMedical Engineering and Science, Tokyo Women’s Medical University, Tokyo, Japan; 4 Long-Term Care Geriatric Health Facility “YUU”, Saitama, Japan; 5 Fujita Medical Instruments Co. Ltd., Tokyo, Japan

**Keywords:** Brain tumor, Continuous tumor resection forceps, Cell isolation mechanism, Flow cytometer, Reflux water

## Abstract

The tumor resection ratio must be improved due the increased possibility of recurrence or malignancy. The purpose of this study was to develop a system that includes forceps with a continuous suction function and flow cytometry to diagnose the malignancy of the tumor for safe, accurate, and effective surgery. A newly developed continuous tumor resection forceps consists of a triple pipe structure, which enables continuous suction of the tumor by integrating the reflux water and suction system. The forceps includes tip opening/closure detection switch to control the adsorption and suction strength when tip is opened and closed. To perform accurate tumor diagnosis using flow cytometry, a filtering mechanism was developed for dehydrating reflux water from continuous suction forceps. In addition, a cell isolation mechanism comprising a roller pump and shear force loading mechanism was also newly developed. By using a triple pipe structure, a significantly larger tumor collection ratio was observed compared to the previous double-pipe structure. By performing suction pressure control with the opening/closure detection switch, inaccurate suction can be prevented. By widening the filter area of dehydration mechanism, it was possible to improve the reflux water dehydration ratio. The most appropriate filter area was 85 mm^2^. By using a newly developed cell isolation mechanism, the processing time can be reduced to less than 1/10 of the original time, keeping the same cell isolation ratio, when compared to the existing pipetting method. Neurosurgery assistance system with continuous tumor resection forceps and a cell separation, dehydration and isolation mechanism was developed. An effective and safe tumor resection, accurate and fast diagnosis of malignancy can be achieved by using the current system.

## Introduction

Brain tumors are a generic term for neoplasms that can be formed intracranially, and in the case of malignancy, tumors invade normal brain tissue [1]. The boundary between a malignant brain tumor and normal brain tissue becomes unclear and it is difficult to surgically remove the tumor completely [2]. However, the residual brain tumor may cause recurrence of surrounding cell or malignant transformation. Therefore, it is necessary to improve the extraction ratio of the tumor to expand the survival ratio. The extraction or resection ratio refers to the percentage of brain tumor that can be removed through the brain surgery. In 2002, Shibuya et al. [3] published a relationship between the resected tumor ratio of a total brain tumor and the 5-years survival ratio. It was found that there was a difference in the 5-years survival ratio when the resected tumor ratio was 95% or more, which is less than 75%. Smith et al. [4] similarly reported that the 5-years survival ratio of patients with a resected tumor ratio less than 90% was 76%, whereas those with a resected tumor ratio of 90% or more had a 5-years survival ratio of 97%.

There is a possibility that the sequelae such as speech disorders or hemiplegia may remain if the normal tissue is injured when removing the brain tumor. Thus, it is necessary to determine the exact extraction range for maximally extracting just the tumor tissue and protecting normal brain tissue [5]. Rapid diagnosis during surgery is a method for extraction range determination that uses a pathological diagnosis result. Intraoperative rapid diagnosis prepares pathological specimens during surgery and diagnoses malignancy of the tumor via a biopsy [6]. However, there are two problems with intraoperative rapid diagnosis namely “procedure of forceps to sample” and “method of collecting pathological specimens.” Pathological specimens are sampled and manufactured by frequent in/out procedures in the surgical field using a surgical instrument called forceps. Hence, it is necessary to repeatedly perform complicated procedures when collecting multiple specimens, which increases the burden on the doctor. Furthermore, the precision of the excision range varies depending on the number of specimens and sampling position, possibly causing leakage of the tumor [7].

An ultrasonic surgical aspiration apparatus (CUSA) manufactured by Cavitron Corporation is available as a surgical instrument for continuous tumor removal. CUSA can crush soft tissue using an ultrasound tissue selection system. However, the apparatus requires that elastic parts such as blood vessels are not broken [8]. On the other hand, it is necessary to secure a wide field of operation to ensure that CUSA vibration is not transmitted to surrounding tissues. The tissue cannot be used for biopsy when aspirated in a state of emulsification. In addition, the previous study on tissue diagnosis considered a brain tissue analysis method that combines CUSA and mass spectrometry. In 2011, Christian et al. [9] proposed a tissue analysis method using a technology called V-EASI. The tissues crushed and emulsified by CUSA are recovered and ionized by the V-EASI technique. The tissue analysis was performing after the data were acquired via mass spectrometry and classified by principal component analysis and linear discriminant analysis. It is necessary to introduce V-EASI technology rather than existing products.

The previous study regarding surgical navigation focused on the navigation system integrating the corticospinal tract and arcuate fasciculus nerve pathways and a navigation system integrating cerebrovascular structures [10–12]. These previous studies have reduced the burden on doctors and improved the tumor resected ratio, indicating the usefulness of the intraoperative navigation system. From this background and previous studies, this study is expected to improve the tumor excision ratio by continuously removing the tumor, increasing the number of pathological specimens and the surgeon performing the tumor extraction based on more diagnostic results. In this study, a support system that enables improvement of the tumor excision ratio by continuous tumor excision and pathological diagnosis was developed. Continuous tumor removal is performed via continuous tumor resection forceps [13], which is a suction mechanism that uses physiological saline (reflux cleaning water), and tumor diagnosis is performed using a flow cytometer. In this paper, we describe in detail the continuous tumor resection forceps, the separation and dehydration mechanism and tumor cell isolation system for fast diagnosis to be introduced into the surgical system.

The content of this paper is distributed as follows; Sect. “Material and methods” discusses the overall system configuration for this study mainly the developed continuous tumor resection forceps, tumor cell separation and dehydration mechanism, and tumor cell isolation mechanism. Sect. “Results” discusses the evaluation of the forceps and the two mechanisms. Sect. “Discussion” discusses the results gained from the evaluation conducted, and lastly, Sect. “Conclusion” concludes the proposed system for this study.

## Material and methods

### System configuration

**Fig. 1:**
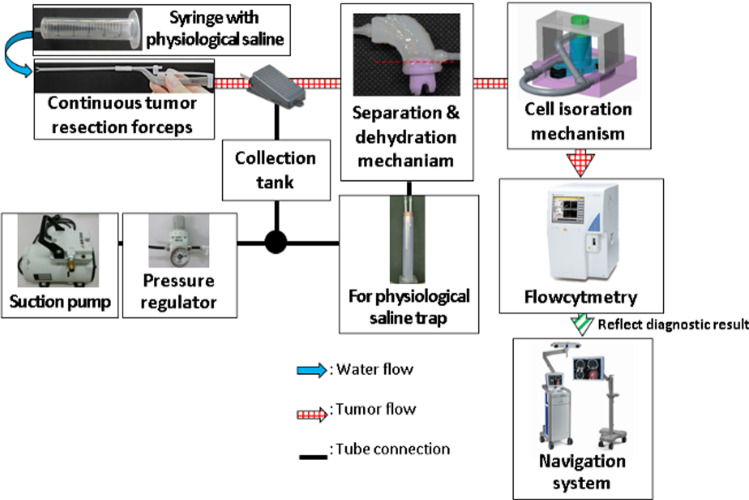
Configuration of continuous brain tumors resection system that include cell diagnosis and navigation system

The surgery assistance system configuration is shown in Fig. 1. The system consists of three main areas which are continuous tumor resection forceps, separation and dehydration mechanism, and cell isolation mechanism. After the tumor is removed using continuous tumor resection forceps, the tumor cell will go through the separation and dehydration mechanism to subtract the reflux cleaning water before collection. After isolating the recovered cells, the cell suspension is automatically passed to the flow cytometer to diagnose the malignancy of the tumor. The diagnosis results are displayed on the navigation system and the extraction range is determined.

The flow cytometer introduced in this system measures the amount of DNA in a cell by staining the cell nucleus with a fluorescent dye. However, if a large amount of water is mixed in the diagnosis, there is a possibility that the concentration of the staining solution will decrease and affect the diagnostic results. Continuous tumor resection forceps under development in this study require dehydration of reflux cleaning water because a large amount of washing water is mixed at the time of tumor collection. Therefore, to perform accurate tumor diagnosis, a separation and dehydration mechanism is developed to separate the reflux cleaning water and the tumor collected in the trap.

### Continuous tumor resection forceps

The continuous tumor resection forceps for this study was developed based on the input from the discussion with two neurosurgeons. The following are the seven requirements for the continuous tumor resection forceps;

1. The outside diameter is equivalent to conventional forceps.
2. The cavity is secured from the tip of the forceps to the root.
3. Bayonet-shaped forceps are used.
4. The opening angle of the tip is equivalent to conventional forceps.
5. When force is applied, the forceps tip is closed.
6. Suction is only executed when the tip is closed.
7. The tip of the forceps is hermetically sealed.

**Fig. 2:**
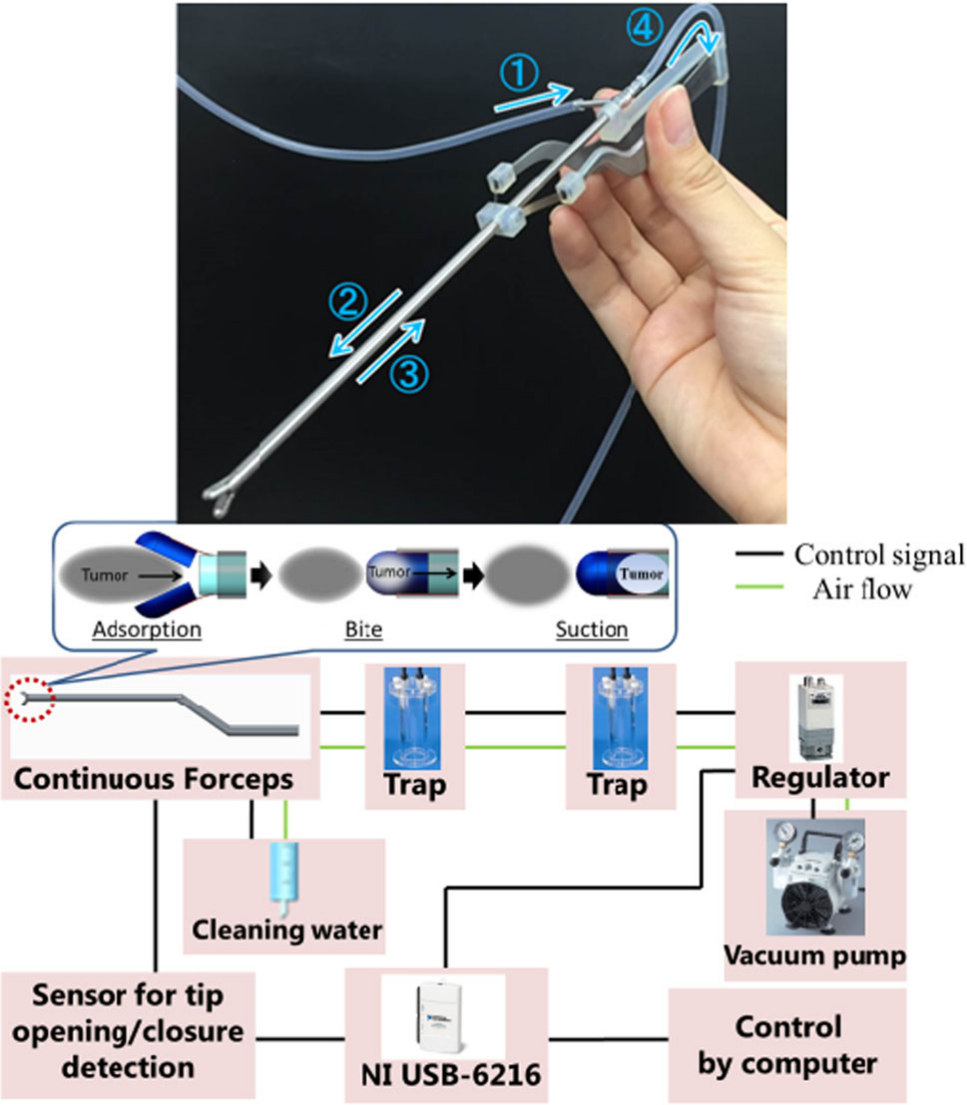
Forceps with continuous suction function for resecting brain tumors and suction system configuration. [13]

**Fig. 3:**
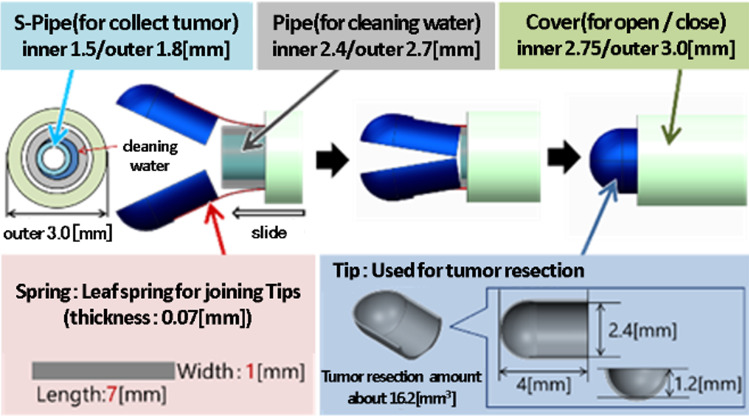
Mechanism of continuous forceps consists of leaf springs, tips, and triple pipe structure

The developed forceps enable continuous removal of tumors by integrating reflux cleaning water and a suction system into conventionally shaped forceps as shown in Fig. 2. The developed forceps do not emulsify the excised tissue because the tumor is resected without using ultrasound. Therefore, it is possible to use the tumor tissue that removed from the forceps as a pathological specimen. The resected tumor is moved to the tumor collection trap (volume of approximately 5 ml) together with reflux cleaning water. After migration, the trap is filled with a recovered tumor and approximately 3 ml of reflux cleaning water. Continuous tumor resection forceps have a triple pipe structure composed of a small pipe (S-Pipe) for collecting a tumor by suction, a middle pipe (Pipe) through which reflux cleaning water flows, and a large pipe (Cover) to open and close the tip of the forceps. In addition, two tips for resecting the tumor are joined by a leaf spring (Spring) (Fig. 3). The tumor collection is aimed at by reflux cleaning water flowing from the middle pipe [13].

After the first forceps prototype was manufactured, the forceps were tested by a neurosurgeon and the following opinions were obtained;

1. Usability is generally good.
2. The opening of the tip is slightly small. There are no problems with narrow spaces, such as pituitary gland surgery.
3. It is difficult to observe whether the forceps are completely closed.
4. It is better if there are variations, such as several lengths of forceps.
5. To evaluate the malignancy via flow cytometry, more specimens are required.

**Fig. 4:**
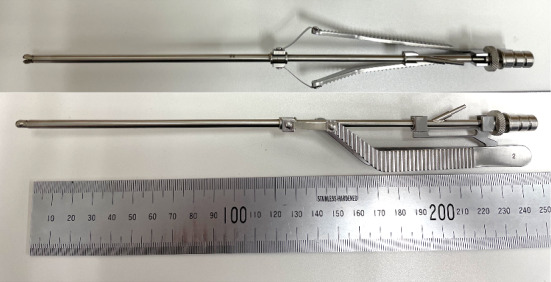
Newly manufactured prototype forceps whose outer diameter and inner diameter of the cup were expanded to increase the amount of specimen taken in one action

**Fig. 5:**
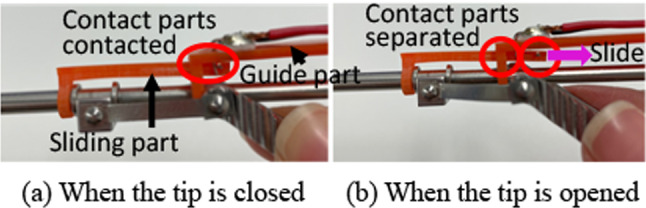
Prototype of tip open-close detection switch detected by the connection of the contact parts

In response to the last requirement, a new prototype was manufactured by Fujita Medical Instruments Co., Ltd. (Fig. 4). The outer diameter of the pipe and inner diameter of the cup were expanded to 4.0 mm and 3.0 mm, respectively, to increase the amount of specimen taken in one action. In addition, an open/close detection switch was integrated into the forceps. It was prototyped by a 3D printer and attached to the latest forceps prototype to assess the amount of extraction (Fig. 5). The open/close detection switch comprises of slide and guide parts. The slide was connected to a cover pipe that slides when opening and closing the tip. The guide part was fixed to the forceps. Contact parts were provided to both parts. When the tip of the forceps is open, the slide slides backward and the contact parts are separated. When the tweezers are pinched and the tip is closed, the slide part moves forward, and the contact parts contact each other. By detecting the contact of the switch, the suction strength is changed by the regulator. When the tip is open, the suction level decreases to −10 kPa. However, when the tip is closed, the suction level increases to −30 kPa.

To evaluate the effect of the triple pipe structure that utilizes the washing water perfusion, the tumor collection ratio was compared with the double-pipe structure by which the middle pipe is excluded and there is no independent route to send reflux water to the tip. Tofu was used as the alternative model and the number of trials was five. The tumor alternative model was resected using continuous tumor resection forceps and collected into a trap by performing suction for 5 s. The water was removed from the collected alternative tumor in the trap. After collecting the tumor alternative model 10 times, the weight difference of the trap before and after collection was measured. The collection ratio was calculated from the ratio of the collection amount collected 10 times without suction.

Similar verification and measurement methods were used to evaluate the influence of suction pressure control on the tumor collection amount using an open/close detection switch. When the switch was used, the suction pressure was controlled to −30 kPa when the tip was closed and −10 kPa when the tip was open. When the switch and control were not in used, the suction pressure was at −30 kPa. Porcine brain was used as a tumor alternative model and five trials were conducted.

### Tumor cell separation and dehydration mechanism

The flow cytometer measures the amount of DNA in a cell by staining the cell nucleus with a fluorescent dye and detecting the amount of fluorescence and the malignancy of the tumor was measured. If a large amount of reflux cleaning water is mixed in the cell nucleus staining, there is a possibility that the concentration of the staining solution will decrease and affect the diagnostic results. Continuous tumor resection forceps collect the tumor and approximately 3 ml reflux cleaning water at the time of tumor collection, diluting the concentration of the staining solution. Therefore, this system requires a mechanism for separating and dehydrating tumors and reflux-cleansing water [14]. The required specifications of the separation and dehydration mechanisms are as follows;

1. Separate and dehydrate in the suction process used for forceps.
2. Both dehydration of wash water and collection of tumors.
3. A target dehydration ratio of 80%.
4. A mechanism that can constantly dehydrate.

**Fig. 6:**
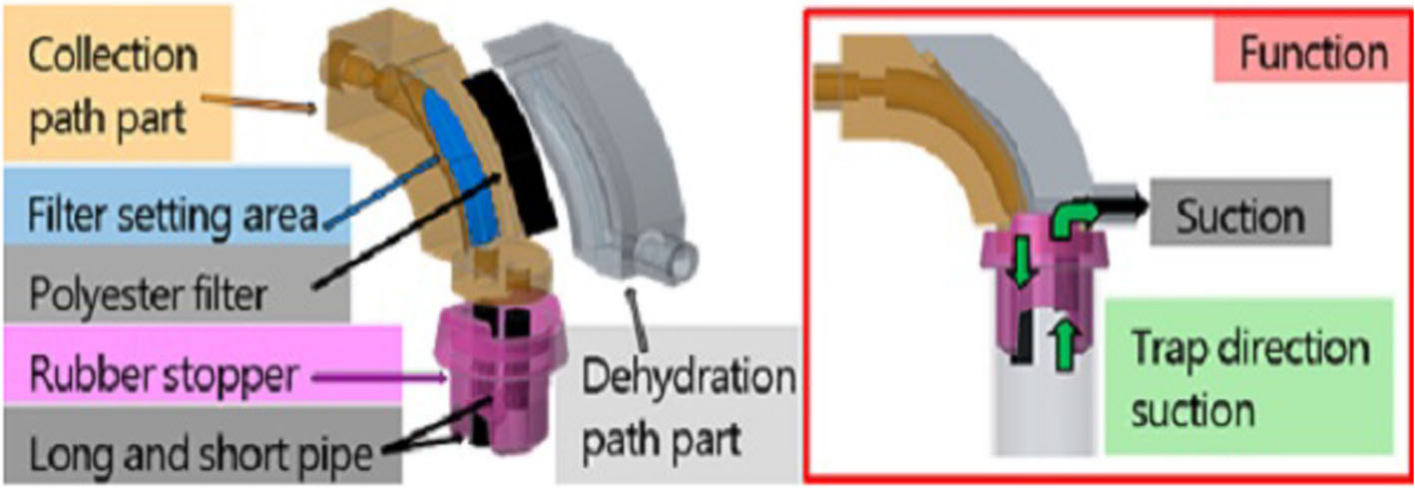
Filter and trap suction mechanisms by which tumor and water are separated by the filter

**Fig. 7:**
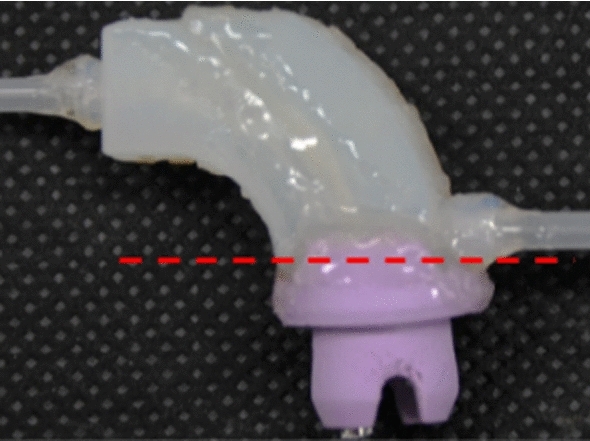
Prototype of mechanism manufactured by 3D printer

The separation and dehydration mechanism has a structure in which a filter is provided outside the arcuate flow path to separate the reflux cleaning water. Figure 6 shows the configuration of the proposed separation and dehydration mechanisms. The mechanism consists of a tumor collection path part, reflux cleaning water dehydration path part, a filter (polyester, opening 60 μm), and two pipes with different lengths. In addition, by providing trap direction suction, the collection ratio of the tumor is improved. By aspirating from the reflux cleaning water dehydration path part, the reflux cleaning water is dehydrated, and the tumor is collected into the trap. The CAD model of the tumor collection path and reflux cleaning water dehydration path part were manufactured using an optical 3D printer and integrated with a filter, rubber stopper, and two pipes (Fig. 7).

**Fig. 8:**
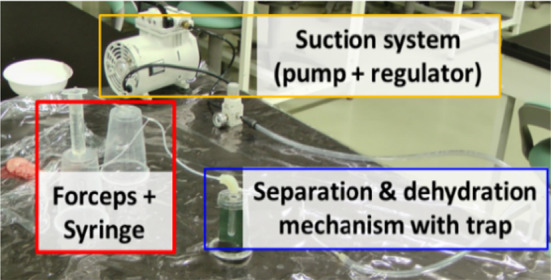
Environment of cell separation and dehydration experiment

The effect of the filter area used for the mechanism of reflux cleaning water dehydration ratio and tumor collection ratio was verified using the prototype mechanism. The experimental environment is shown in Fig. 8. Porcine brain was used as a tumor alternative model. Fifty trials were conducted for the reflux cleaning water dehydration ratio and five trials for the tumor collection ratio. Four types of filter areas were used for verification: 56.78, 70.76, 85, and 100 mm^2^. The tumor alternative model was resected with continuous tumor resection forceps and suction was performed for 5 s. The reflux cleaning water was dehydrated via the separation and dehydration mechanism and the tumor alternative model was collected in the trap. The ratio of dehydration was calculated by measuring the amount of reflux cleaning water flowing from the syringe and the amount of reflux cleaning water in the trap. The collection would be considered successful if the tumor alternative model did not remain in the separation and dehydration mechanism.

### Tumor cell isolation mechanism

**Fig. 9:**
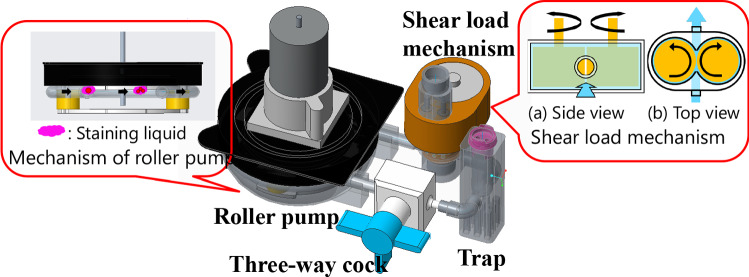
Configuration of cell isolation mechanism composed by roller pump, shear load loading mechanism, three-way cock and trap

**Fig. 10:**
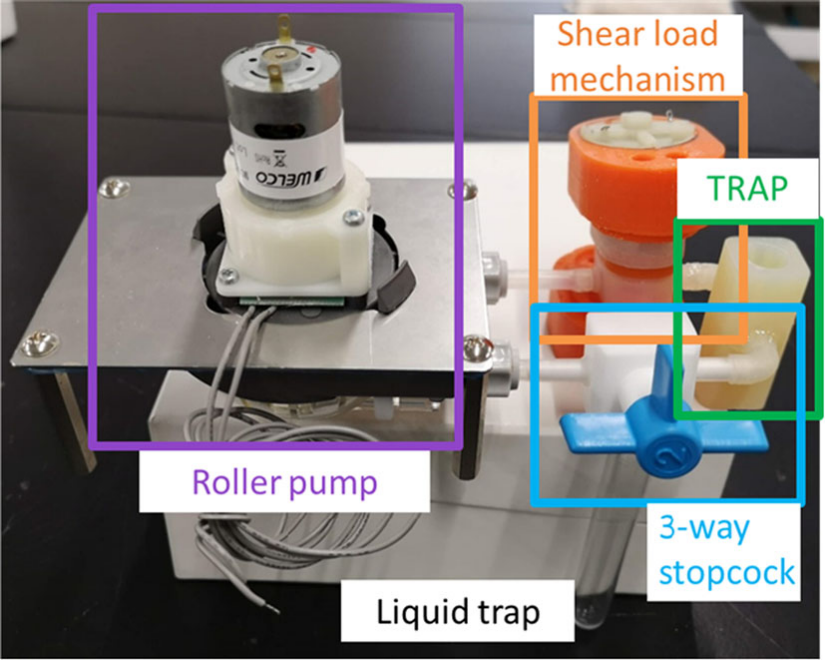
Overview of manufactured cell isolation mechanism

Tumor malignancy was measured by the quantity of DNA, via flow cytometry. For accurate diagnosis, cells should be isolated. In the existing method, it takes approximately 10 min from removal to diagnosis and approximately 6 min to isolate cells by pipetting. The configuration of the newly developed cell isolation mechanism [15] is illustrated in Fig. 9. An overview of the manufactured mechanism is presented in Fig. 10. The isolator comprises a roller pump and a shear force loading mechanism. In the shear force loading mechanism, a shear force is applied by forcing a solution that contains reagents and cells between two counter-rotating rollers, driven by a motor and gears. The solution was circulated using a roller pump. For accurate malignancy diagnosis, the solution used in the previous session must not remain. A new outlet for the solution was created separately from the inlet using a three-way cock to improve the solution recovery rate and cleaning performance [16].

The ability of the newly developed cell isolation mechanism to reduce the cell isolation time was evaluated and compared with the pipetting method. As a tumor substitute model, 3 mm^2^ of porcine brain and 3 ml of DNA staining solution were injected into the device and cells were isolated under three conditions of 0.5, 1.0, 2.0, 3.5 and 5.0 min. The single-cell nucleus ratio was measured using flow cytometry. Five measurements were performed for each condition. Lastly, experiments were conducted to test the device’s cleanability. First, 3 ml water with red food coloring at a concentration of 1% was poured into the device. The transition of absorbance ratio compared with the original density was measured by repeating the process of rotating and adding 3 ml water.

## Results

### Evaluation of forceps on the influence of triple pipe structure by tumor collection ratio

**Fig. 11:**
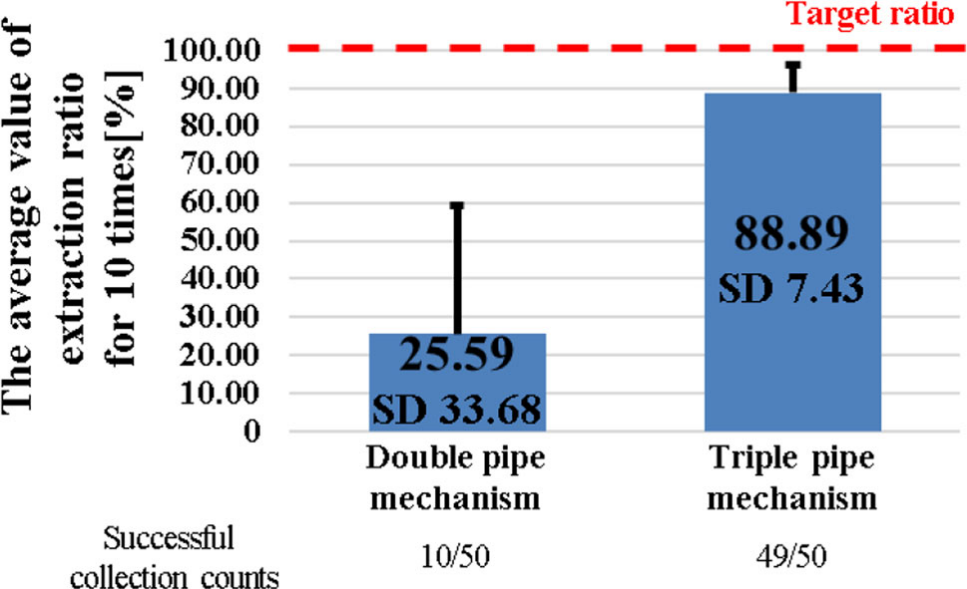
Comparison of suction performance for different forceps with double and triple pipe structure

The results of suction performance between double and triple pipe structures are shown in Fig. 11. It was found that continuous tumor resection forceps with triple pipe structure gained a higher collection ratio than the double-pipe structure. As shown previously in Fig. 9, it was difficult for the double-pipe structure to perfuse with reflux cleaning water and the collection ratio was lower than the target collection ratio.

### Evaluation of the effect of suction pressure control by opening/closing detection switch

**Fig. 12:**
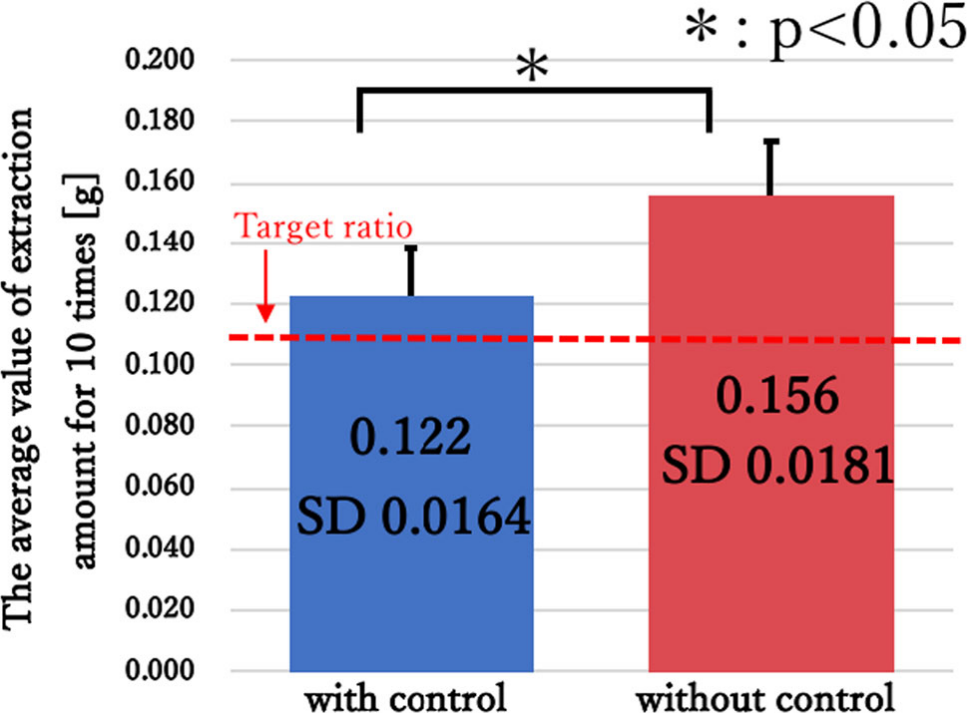
Comparison of resection and suction performance with and without suction control

Figure 12 shows the weight of the collected specimen over 10 trials, with and without control. The verification results show that when the suction pressure was controlled, the collection amount was almost equal to the collection target value. On the other hand, when suction pressure control was not in used, the collection amount significantly exceeded the target value.

### Evaluation of tumor cell separation and dehydration mechanism

**Fig. 13:**
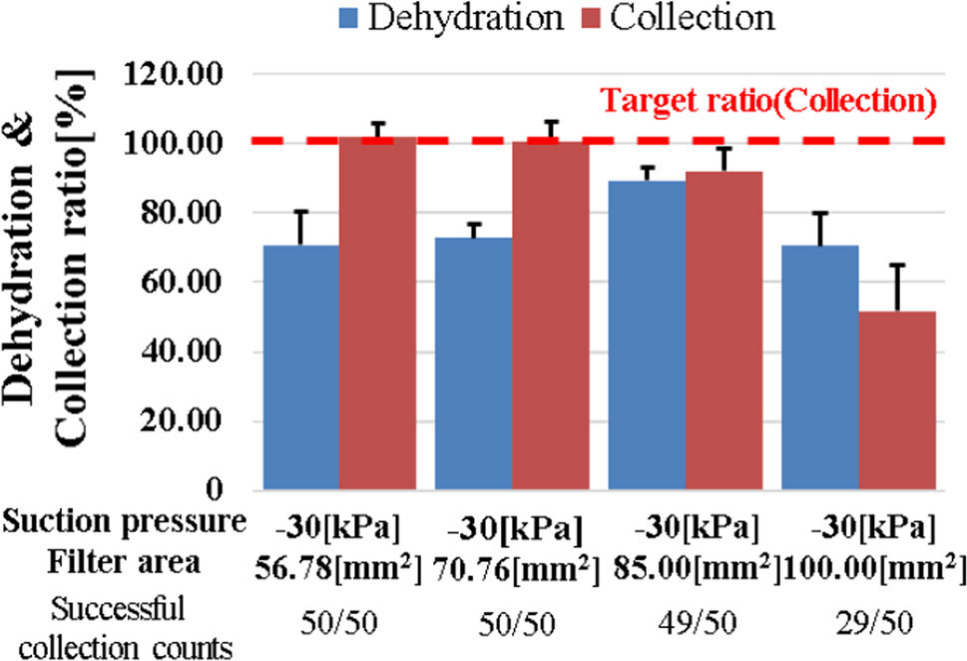
Results on the effect of filter area for cleaning water dehydration and tumor collection ratio

The verification results of the effect of the filter area used for the mechanism are shown in Fig. 13. The model with a filter area of 85 mm^2^ obtained the highest dehydration ratio of 89.30% with a standard deviation of 3.65%. When the filter area was 100 mm^2^, the dehydration ratio dropped to 70.57%. When the filter area was 85 mm^2^ or more, the target collection ratio decreased.

### Evaluation of cell isolation mechanism

**Fig. 14:**
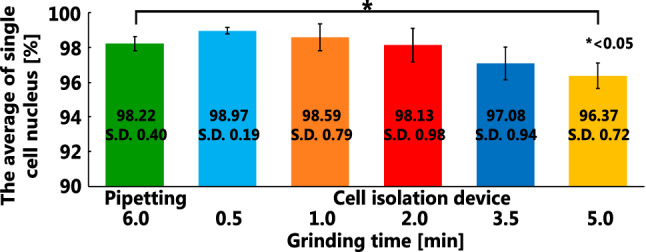
Results of single-cell isolated ratio when grinding time was changed and compared with the existing pipetting method

The evaluation results of single-cell isolation ratio are presented in Fig. 14. With a cell isolation time of 0.5 min, 99% of the single-cell nucleus ratio, which was equivalent to the existing pipetting method, was obtained. When the grinding time was increased to 5 min, the single-cell ratio decreased to 96%. In addition, a cell nucleus concentration of 50 cell/l or more, which is necessary for diagnosis, was obtained. In addition, by creating a new outlet using a three-way cock, collecting the solution while rotating the roller pump became possible. As a result, the solution collection rate improved from 40% to approximately 75%.

**Fig. 15:**
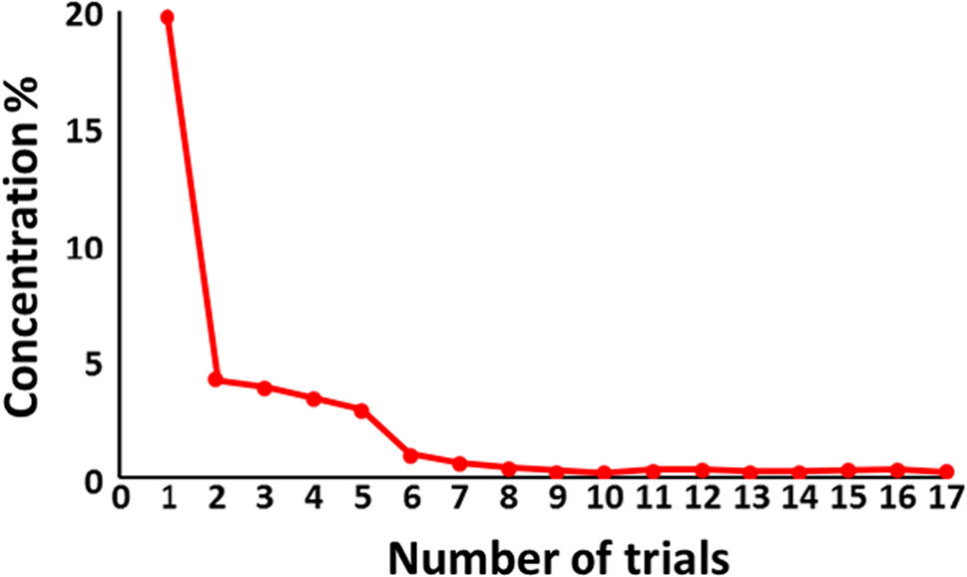
Transition of absorbance ratio compared with the original density when number of cleaning trials were increased

Lastly, the device’s cleanability test results are presented in Fig. 15. After eight washes (24 ml), the absorbance was 1/100 or less and almost no change occurred. The same condition was maintained up to 17 washes and the results were similar to eight washes.

## Discussion

### Continuous tumor resection forceps

Using the triple pipe structure provided with reflux cleaning water perfusion, the collection ratio was increased by the perfusion of washing water. The reason for the collection ratio of the triple pipe structure being lower than the target collection ratio is that the tumor alternative model was finely pulverized via suction with a vacuum pump, and it was difficult to collect a small tumor alternative model. Based on the collection ratio of the double-pipe structure and triple pipe structure, it was suggested that triple pipe structure forceps with reflux cleaning water perfusion is more effective for tumor collection, as the collection ratio of the triple pipe structure is significantly larger (*p* < 0.05).

The collection volume exceeded the collection target ratio without suction pressure control because the tumor alternative model was erroneously aspirated when the tip of the forceps was open. These results suggest that by controlling the suction strength by opening and closing the tip, inaccurate aspiration of the tumor can be prevented, and safe removal of the tumor becomes possible.

### Tumor cell separation and dehydration mechanism

The number of tumors with successful tumor collection times was less than 100% when the filter area was 85 mm^2^ or more because the suction force received by the filter from the tumor alternative model increases as the filter area becomes wider. Therefore, the tumor alternative model became easily caught by the filter and the collection ratio became smaller. The model with a filter area of 85 mm^2^ was nearest to the required specification as the dehydration rate was highest and collection only failed once. As the result showed that missing number of cells are low and dehydrated effectively, the accuracy of diagnosis will not be influenced by the developed mechanism.

### Tumor cell isolation mechanism

Using the newly developed cell isolation mechanism which comprises a roller pump and shear force loading mechanism, the processing time can be reduced to less than 1/10 compared to the existing pipetting method. It was proved that the circuit could be cleaned after eight washing processes (24 ml). As the tumor cell includes more DNA compared to normal cell, we can diagnose the percentage of tumor cells included in the specimen. And if the single separation ratio is higher, we can get higher accurate result. By reducing the processing time, fast and frequent diagnosis can be realized. Thus, neurosurgeons can get the region of tumor faster and more accurately.

Further improvements of cell isolation mechanism should be attempted using computational fluid flow analysis and washing process should be automized for reducing the time. Besides, an automatic transfer system that can input brain tumors from separation and dehydration mechanisms and outputs isolated cells to the flow cytometer should be developed.

## Conclusion

In this study, the surgery assistance system consists of the continuous tumor resection forceps, a cell separation, dehydration, and isolation mechanism had been constructed and developed. The developed surgical instruments perform continuous tumor removal without requiring potentially problematic procedures when collecting pathological specimens. Continuous tumor resection forceps have a triple pipe structure and continuous suction of the tumor is possible by integrating reflux cleaning water and a suction system. By comparing the performance of continuous tumor excision forceps with a double-pipe structure, it was possible to obtain a significant tumor collection ratio. In addition, it is possible to prevent inaccurate aspiration by controlling the suction pressure with an open/close detection switch integrated into the forceps and to perform safe tumor resection by controlling the adsorption and suction strength. To perform accurate tumor diagnosis using a flow cytometer, we developed a mechanism for dehydrating, approximately 3 ml cleaning water flowing from continuous tumor resection forceps. The separation and dehydration mechanism has a filter on the outside of the arcuate flow path and comprises five parts. By widening the filter area, it was possible to improve the reflux cleaning water dehydration ratio. With the newly developed cell isolation mechanism, which comprises a roller pump and shear load applying mechanism, the processing time can be reduced to less than 1/10 of the original time.

As for the future work, it will be necessary to redesign in detail, including parts manufactured by 3D printers such as open/close detection sensors, and to perform endurance tests on prototype forceps. Regarding the separation and dehydration mechanism, although the requirement for the reflux cleaning water dehydration ratio was satisfied, the tumor collection ratio should be improved. Furthermore, it is necessary to verify the usability of the system, assuming an actual surgical environment, and verify whether accurate intraoperative rapid diagnosis is possible by using a system that integrates continuous tumor resection forceps with an open/close detection switch, separation and dehydration mechanism, cell isolation system, and flow cytometry. In addition, a malignance-level feedback system to the navigation system by which doctors can operate safely and effectively should be developed.
